# Development of Vinpocetine-Loaded Nasal Polymeric Micelles via Nano-Spray-Drying

**DOI:** 10.3390/ph16101447

**Published:** 2023-10-12

**Authors:** Bence Sipos, Gábor Katona, Flóra Mária Szarvas, Mária Budai-Szűcs, Rita Ambrus, Ildikó Csóka

**Affiliations:** Institute of Pharmaceutical Technology and Regulatory Affairs, Faculty of Pharmacy, University of Szeged, Eötvös Street 6, H-6720 Szeged, Hungary; florka98@gmail.com (F.M.S.); budai-szucs.maria@szte.hu (M.B.-S.); ambrus.rita@szte.hu (R.A.); csoka.ildiko@szte.hu (I.C.)

**Keywords:** polymeric micelle, nasal delivery, nano-spray-drying, solubility enhancement, permeability enhancement, mucoadhesive polymer

## Abstract

In this present formulation study, vinpocetine-loaded nano-spray-dried polymeric micelles were developed via nano-spray-drying. Three different mucoadhesive excipients were applied in the studies, namely chitosan, hyaluronic acid and hydroxypropyl methylcellulose. In all cases, the formulations had a proper particle size and drug content after drying with spherical morphology and amorphous structure. After rapid dissolution in water, the polymeric micelles had a particle size around 100–130 nm, in monodisperse size distribution. The high encapsulation efficiency (>80%) and high solubilization (approx. 300-fold increase in thermodynamic solubility) contributed to rapid drug release (>80% in the first 15 min) and fast passive diffusion at simulated nasal conditions. The formulated prototype preparations fulfilled the demands of a low-viscosity, moderately mucoadhesive nasal drug delivery system, which may be capable of increasing the overall bioavailability of drugs administered via the auspicious nasal drug delivery route.

## 1. Introduction

Nasal drug administration presents numeral challenges due to unique anatomical and physiological characteristics of the nasal cavity; however, it holds even more advantages. Due to the viewpoint of therapeutic considerations, it can be a proper choice to achieve rapid drug release and absorption compared to other conventional drug administration routes [1,2,3]. Most drugs are absorbed through passive diffusion, which requires a nasal dosage form, where there is only a limited matrix system forming an obstacle to free diffusion. The other main advantage is that via the nose-to-brain drug delivery route, the active substances can reach the central nervous system, bypassing the blood–brain barrier [4,5]. This can also be controlled and enhanced via nanocarriers, such as polymeric micelles.

Polymeric micelles are nanoparticle-sized, self-assembling structures from the spontaneous self-association of amphiphilic block co-polymers in aqueous solutions. These unique core–shell structures can provide higher solubilization capacity compared to other nanocarriers alongside with higher stability. Their versatility and ability to carry a variety of active substances make them promising candidates in the case of enhancing the efficacy and safety of certain drugs [6,7,8]. Their nose-to-brain, direct transport is controlled by their particle size alongside with their surface-charge-mediated absorption profile. But rapid absorption across the nasal mucosa can also be beneficial in the case of brain targeting since their shell structures can defend the active substance from degradation in the blood for a longer time [9,10,11,12].

Viscosity and mucoadhesivity are two vital parameters regarding nasal drug delivery. Balancing these parameters is essential since it influences both the therapeutic efficacy and the overall bioavailability of the administered drug. The most important rule is that the applied excipients and their concentration should meet the product target quality criteria. Regarding a drug delivery system with rapid drug release and absorption, it is beneficial to reduce their concentration to the minimum possible amount. In this way, they would not be responsible for the formation of a thick matrix system where passive diffusion is inhibited. However, the formulation should be resided in the nasal cavity for at least 15 min since the natural elimination mechanism, the mucociliary clearance, can take place within this time frame [13,14,15]. Commonly applied excipients include chitosan (Chit), which is a polysaccharide-type biopolymer derived from chitin and it is widely utilized in drug delivery due to its ability to enhance drug absorption and facilitate controlled release of therapeutic agents [16]. Hyaluronic acid (HyA) is also a naturally occurring polysaccharide, holding viscoelastic properties contributing to increased viscosity and extending drug residence time [17]. Another group of the nasal mucoadhesive excipients includes semi-synthetic cellulose derivates, such as hydroxypropyl methylcellulose (HPMC), also applied for the same purposes [18]. All three mentioned nasal drug delivery system excipients are biocompatible and biodegradable [19].

The difference between these polymers not only lies in the physicochemical characteristics, but rather how they affect drug permeation through biological membranes. Hyaluronic acid is known to be a biocompatible permeability enhancer through the paracellular pathways [20,21]. Chitosan also plays a prominent role in absorption enhancement. Chitosan can increase cell permeability, affecting paracellular and intracellular pathways of epithelial cells. The main advantage in the case of chitosan is that this mechanism-modifying property is reversible and it does not affect the normal cell functions [22,23]. HPMC and its derivatives are generally not applied for their permeability enhancement function, but rather its function as a viscosupplementation [24].

The aim of the series of experiments was to demonstrate the development of a nasal drug delivery system with three different biocompatible nasally mucoadhesive polymers. The added value of the mucoadhesive polymers lies in the increase in residence; however, it was aimed to develop low-viscosity formulations, not to hinder further rapid drug release kinetics. To add further value, the colloidal and in vitro behavior of polymeric micelles was implemented in the research design, first to solve the problem of water solubility regarding vinpocetine (VP), and later to ensure a large and rapid release and permeability during the nasal residence time. To ensure stability to the product, nano-spray-drying was chosen as a scalable, one-step process. As a result of the development, a prototype preparation was formed, which can be suitable for the administration of active substances affecting the central nervous system, as well as VP, used as a model compound in this case, through the nasal administration route.

## 2. Results

### 2.1. Optimization of VP-Loaded Polymeric Micelles

The optimization of VP-loaded polymeric micelles was performed via a two-factor, three-level factorial design, where the effect on average hydrodynamic diameter (Z-average), as micelle size and polydispersity index (PdI) of the amount of the applied polymeric-micelle-forming polymers Poloxamer 188 (P188) and Soluplus^®^ (SP), was investigated. The results from the factorial design can be seen in Table 1.

**Figure 1:**
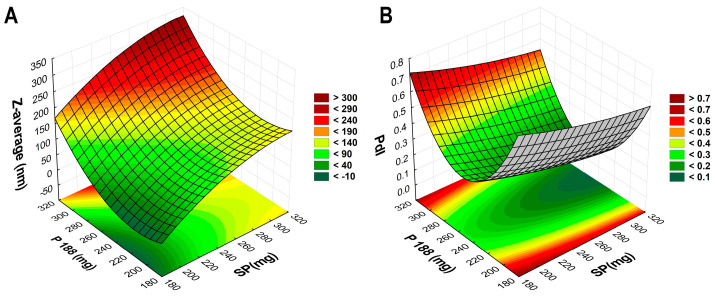
Three-dimensional surface plots based on the 2-factor, 3-level factorial design. (**A**) demonstrates the effect of the polymer amounts on Z-average, whilst (**B**) demonstrates the effect on PdI.

Based on the results in Table 1, 3D surface plots (Figure 1) and polynomial equations (Equations (1) and (2)) were constructed. Since no significant factor was found during the analysis, the run with the lowest PdI and proper particle size was applied later during the addition of the nasal excipients: to encapsulate 25 mg of VP, 250 mg of P188 and 300 mg of SP would be applied.

(1) $$Z-\mathrm{average}=136.66+56.06x_{1}+9.37x_{1}^{2}+57.36x_{2}-20.42x_{2}^{2}$$

(2) $$\mathrm{PdI}=0.329-0.066x_{1}-0.006x_{1}^{2}-0.018x_{2}-0.117x_{2}^{2}$$

### 2.2. Solid-State Characterization of the Optimized Formulations

#### 2.2.1. Particle Size Determination via Laser Diffraction

The average particle size of the initial VP and the nano-spray-dried formulations was measured via laser diffraction and the resulting particle size and calculated Span values can be found in Table 2. 

Based on the measurement, the crude VP has a heterodisperse size distribution with a larger D[0.5] value compared to the other formulations’ average particle size as well. After the spray-drying process, VP can either be encapsulated into the polymeric matrix or the unencapsulated part could be found on top of the D-trehalose dihydrate (D-TRE) particles. Regarding all formulations, an average particle size around 7 µm was achieved in monodisperse distribution. Since the D[0.9] values are relatively close to the D[0.5] values, it can be stated that aggregation did not occur in a significant manner during the drying process. Note that this technique also disperses the particles when sucked into the measurement unit with a high shear rate, which can also break up any aggregates [25]. Since the final formulation would be a nasal liquid, any excess formation is irrelevant unlike in the case of nasal powders. The possibility of melt and dried aggregate formation of the applied co-polymers can also be omitted.

#### 2.2.2. Drug Content

In the next step, the drug content of the solid-state, spray-dried formulations was determined. Followed by the dissolution of the samples in the purified water–methanol mixture, the quantification was performed via high-performance liquid chromatography (HPLC). The theorical and the measured drug content can be found in Table 3. 

The measured drug content is close to the theoretical one; therefore, a robust and well-producing production method was applied to achieve the desired formulation. Based on the measured drug content values, the samples were diluted with purified water to the extent required to gain a VP micellar solution of 250 µg/mL for further liquid-state investigations under quantitative control via HPLC.

#### 2.2.3. Morphology

**Figure 2:**
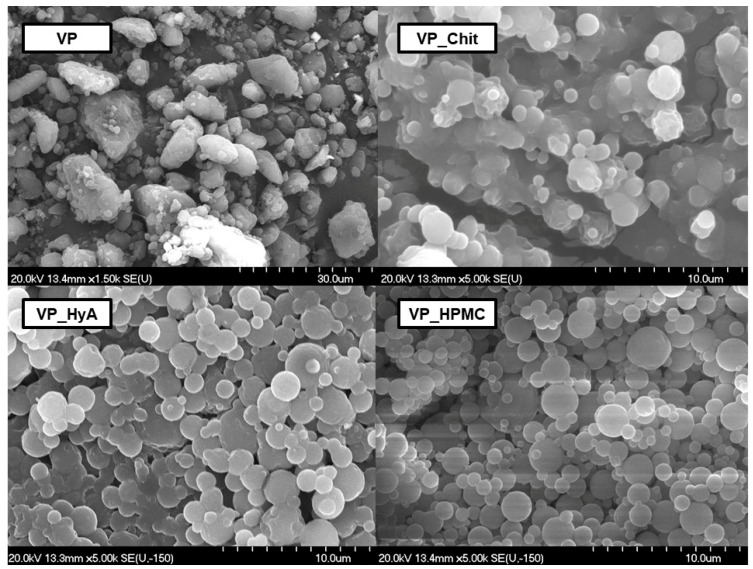
Scanning electron microscopic captures of the initial VP and the three mucoadhesive-containing nano-spray-dried formulations.

Morphological studies were performed via scanning electron microscopy and the image of the captured particles can be found in Figure 2. 

The initial VP has a crude and irregular shape and edge system and a heterodisperse particle size distribution. Aggregation and built-up structures can also be found. Regarding the VP_Chit formulation, spherical particles are formed; however, cluster formation occurred. The same can be claimed about the other two formulations, VP_HyA and VP_HPMC, however, to a smaller extent. This can be explained with the high adhesivity amongst the co-polymers applied alongside with the excipient’s own high-adhesive nature as well. Since all co-polymers and mucoadhesive excipients are water-soluble, these clusters would break easily after dispersion in water. Based on these results, the dispersion time was measured under constant stirring at 750 rpm at ambient temperature. The slowest dispersion was experienced in the case of VP_Chit formulation with a time value of 31.5 s, followed by VP_HPMC with 25.1 s and VP_HyA with 14.3 s. This confirmed that even though cluster formation occurred, it did not affect the dispersion of the formulations to an extent where it would cause inconvenient agitation prior to administration as an ex-tempore formulable nasal liquid. 

#### 2.2.4. X-ray Powder Diffraction Studies

**Figure 3:**
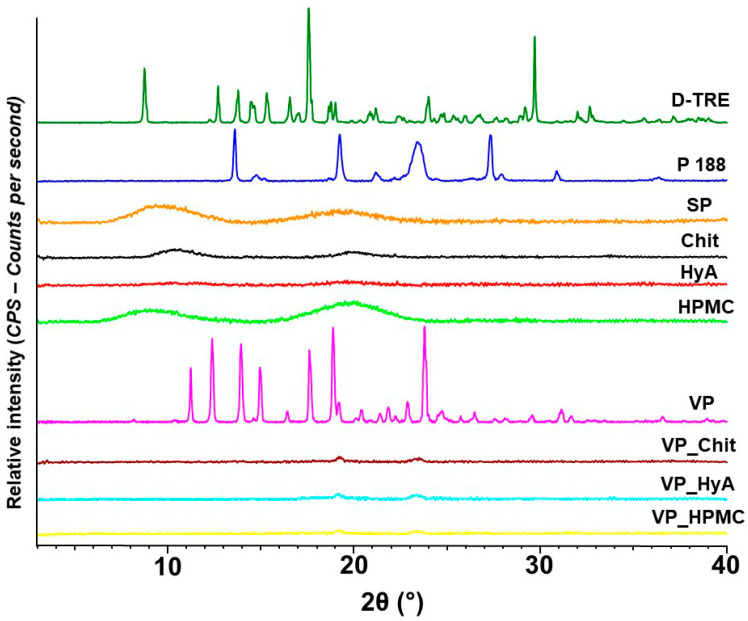
X-ray powder diffractograms of the individual components and the formulations. From top to bottom: D-trehalose dihydrate (D-TRE), Poloxamer 188 (P 188), Soluplus^®^ (SP), Chit (chitosan), HyA (hyaluronic acid), HPMC (hydroxy-propyl methyl-cellulose), VP (vinpocetine) and the VP_Chit, VP_HyA and VP_HPMC nano-spray-dried formulations.

To characterize the crystallinity of the nano-spray-dried formulations, XRPD measurements took place and the acquired diffractograms can be found in Figure 3.

Prior to the drying process, it can be seen that all mucoadhesive excipients and SP are amorphous in nature. D-TRE and VP have a very sharp and exact crystalline structure and a partial crystalline nature can be found in the diffractogram of Poloxamer 188 as well. After the drying process, all three formulations are amorphous in nature and no crystalline peak can be observed. This concludes the successful encapsulation of VP into the polymeric matrix. D-TRE is also a well-known spray-drying excipient, which would achieve an amorphous structure after spray-drying. The same can be claimed about D-TRE after freeze drying as well [26]. The amorphous nature and the small particle size values of the nano-spray-dried formulations would all lead to the quick dispersion in aqueous media as explained before. 

### 2.3. Liquid-State Characterization of the Optimized Formulations

#### 2.3.1. Micelle Size, Size Distribution and Zeta Potential

Based on the results of the dynamic light scattering measurements (Table 4), all dispersed formulations had a micelle size corresponding to the average of 20 to 200 nm of polymeric micelles. The PdI values were also of proper value, below 0.300, meaning uniform absorption could be experienced in the monodisperse formulations. The mucoadhesive excipients all faded the surface charge properties of the blank polymeric micelles (−14.25 ± 1.85 mV), meaning that they most likely cover the nanoparticles’ surface. In the case of VP_Chit, a high positive value was obtained corresponding to the surface charge properties of pure chitosan. The positive charge is also useful in the case of nasal administration, as the nasal mucosa can be characterized by a negative surface, meaning that stronger ionic interactions could also take place besides the physical ones. In the case of VP_HyA and VP_HPMC, the absolute value of the surface charge decreased, meaning lower colloidal stability could be expected as the particle–particle repelling forces have low values. Generally speaking, polymeric micelles tend to take up two different routes based on the total surface charge. In case of VP_Chit as the positively charged polymeric micelle, it can be called a mucoadhesive micelle where the carrier would be embedded into the mucus layer and passive diffusion would be the main force for drug permeation. VP_HyA and VP_HPMC as negatively charged (VP_HPMC can be characterized with no surface charge as well, due to the extremely low value) polymeric micelles are called mucopenetrating micelles, where the mucus layer allows carrier-intact transcytosis or paracellular transport [27]. 

#### 2.3.2. Encapsulation Efficiency and Thermodynamic Solubility Measurement

The encapsulation efficiency reflects the amount of the active pharmaceutical ingredient (API) in a dissolved form compared to the whole system itself. The highest encapsulation efficiency (EE%) value was obtained in the case of VP_Chit, compared to VP_HyA and VP_HPMC (Table 5). The mucoadhesive-excipient-free formulation had an EE% of 80.12 ± 1.7%, meaning that excess solubilizing was obtained in the case of the addition of the mucoadhesive excipients. Chitosan itself is a common biopolymer applied for micelle formation besides its coating property on the surface of nanoparticles. Thus, the significantly increased EE% (compared to excipient-free polymer micelle formulation; *, *p* < 0.05) can be validated. The same can be claimed about the micelle forming properties of HyA, where the literature states that it can also be applied alongside with other surfactants as well. HPMC is a classic cellulose-derived polymer, which barely contributes to the solubility enhancement in nasal liquid formulations; however, as a strong mucoadhesive polymer, it is also advised to apply it. Aiming for the burst type of drug release and permeation properties, based on the micelle size, the zeta potential and the EE% measurements, it would be advised to choose the VP_Chit formulation compared to the others. This tendency also shows in the thermodynamic solubility measurement, where the highest solubility enhancement was achieved in the case of the VP_Chit spray-dried formulation by almost 300-fold. 

#### 2.3.3. Viscosity Measurement

**Figure 4:**
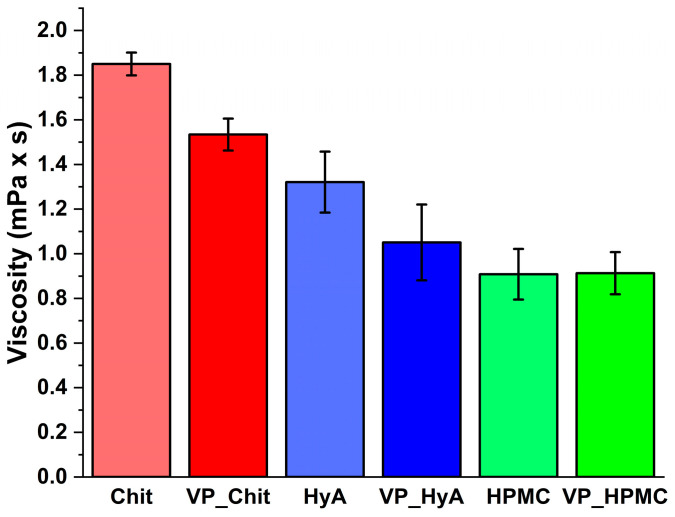
Dynamic viscosity of the formulations at 50 1/s compared to pure mucoadhesive polymer solutions in the same concentration as in the formulations. Data are presented as average ± SD (*n* = 3).

The values of dynamic viscosity can be seen in Figure 4, where a mucoadhesive polymer solution with the same concentration was used as a reference for the formulations. This low viscosity can be beneficial in several ways, as they can be easily sprayed from a nasal spray pump or applied from a nasal drop dispenser. Since polymeric micelles are generally characterized by a fast absorption profile, they do not cause a significant problem due to possible runoff during the average nasal residence time (i.e., 15–20 min). Furthermore, the low viscosity does not result in a feeling of discomfort from the patient’s point of view, and the nasal cavity does not perceive it as a foreign substance. The low viscosity also indicates that the applied low-concentration polymers do not form a diffusion barrier in the administered system, so a free and rapid drug release of the active substances is expected. In the case of the pure Chit solution, a significant difference can be found compared to the VP_Chit formulation (*, *p* < 0.05), which can be explained with the fact that the applied active substance can change the hydration profile of the applied polymers. A slight decrease can also be seen in the case of VP_HyA; however, it is not significant (n.s., *p* > 0.05).

### 2.4. Nasal Applicability Studies

#### 2.4.1. In Vitro Mucoadhesion Study

**Figure 5:**
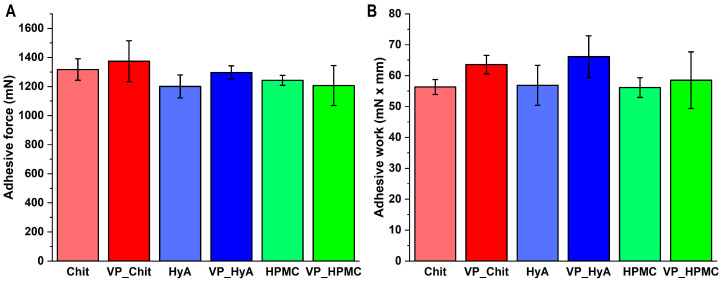
Mucoadhesive force (**A**) and mucoadhesive work (**B**) values of the liquid-state formulations compared to pure mucoadhesive polymer solutions in the same concentration as in the formulations. Data are presented as average ± SD (*n* = 5).

The in vitro mucoadhesion measurements under nasal conditions showed that the formulations are characterized by moderate mucoadhesive strength and work (Figure 5). Compared to previous results by our research group, the values are higher compared to nasal polymeric micelle formulations without any additive mucoadhesive excipients. However, compared to higher concentrations of these excipients, the values are naturally lower. However, this fulfills the objectives set in order to exert the mucoadhesive effect of these substances at a low concentration, but they do not adhere to the nasal cavity to such an extent that it would inhibit the rapid release and absorption of the active substance.

#### 2.4.2. In Vitro Drug Release Study

**Figure 6:**
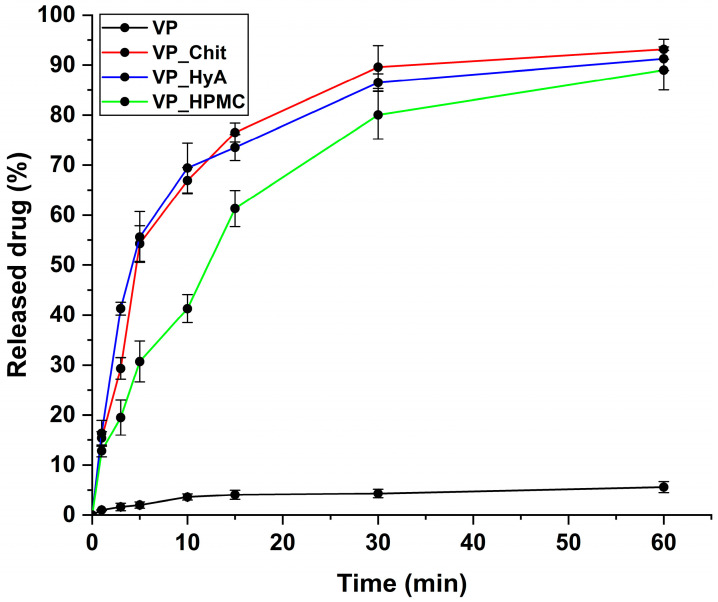
In vitro drug release curves at simulated nasal conditions. The initial VP was compared to the liquid-state, dissolved nano-spray-dried formulations. Data are presented as average ± SD (*n* = 3).

An in vitro drug release study was performed under simulated nasal conditions. The criterion was that the fast release of the active substance must be ensured. Pure VP was applied as a reference, and it is clearly visible on the drug-release–time curves (Figure 6) that all the formulations increased the drug release compared to it. The most critical time window in this case is the first 15–20 min, as this is how long the formulation resides on the nasal mucosa before mucociliary clearance would eliminate it. All three products can be described with rapid drug release and the burst effect can be seen, meaning that a large amount of the active substance is released in a short period of time. The rapidest and highest drug release was achieved via the VP_Chit formulation due to the highest solubilization and encapsulation efficiency experienced in prior measurements.

In Table 6, the results of the kinetic calculations can be seen. The Higuchi kinetic profile fitted the best in all cases of the three formulations, which is typical for polymeric micelles with a rapid drug release and burst effect, as the shape of their dissolution curves is like a root function and in our case, the diffusion from the matrix system is negligible due to the applied low concentrations [23].

#### 2.4.3. In Vitro Nasal Diffusion Study

**Figure 7:**
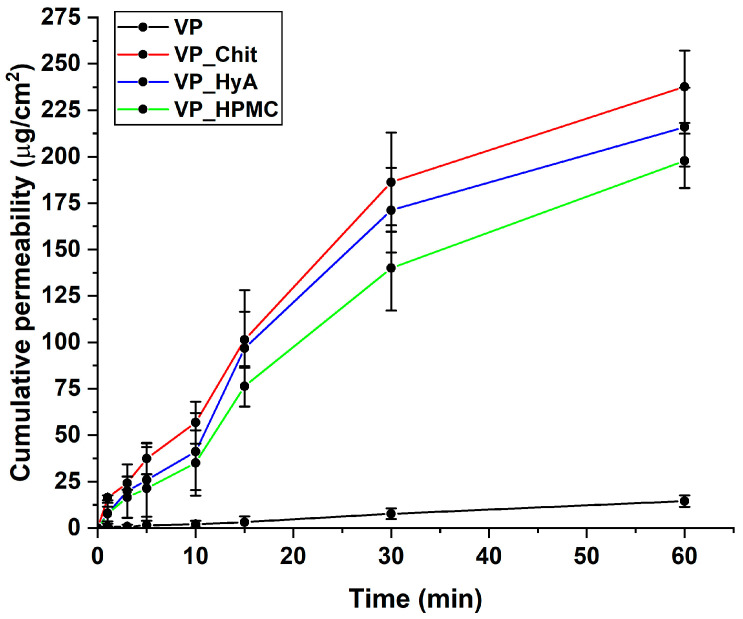
In vitro drug release curves at simulated nasal conditions. The initial VP was compared to the liquid-state, dissolved nano-spray-dried formulations. Data are presented as average ± SD (*n* = 3).

During the in vitro nasal diffusion measurements, passive diffusion under nasal conditions was modeled. The cumulative-permeability–time curves show that compared to the initial VP, the formulation provided approximately 15–20 times of the penetration of VP in the case of the three formulations (Figure 7). The best results are obtained in the case of VP_Chit, which can be deduced from the results of the in vitro drug release study as well. The calculated parameters are in Table 7, and it also supports the enhanced permeability profile of the nano-spray-dried formulations.

## 3. Discussion

As the first step of the series of experiments, the VP-loaded polymeric micelles were optimized without the mucoadhesive excipients using the nano-spray-drying technique. As a result of the factorial design, it became clear that appropriate polymers were selected, as they were able to solubilize VP to a large extent, while producing polymeric micelles with the appropriate micelle size and size distribution. The three additive polymers did not affect these characteristics. The difference between the surface charges also excellently appeared; in the case of chitosan, it was positive, while in the case of the other two polymers, the totality of the system had a negative sign, which can also affect the method of absorption.

The content of the active substance was easily controlled and reproducible during the experiments, with little loss to the dried powders. Based on the morphological examination, it can be confirmed that the encapsulation was successful; spherical particles of the appropriate size were obtained in the case of the products containing chitosan and hyaluronic acid, while slight aggregation was observed in the case of the HPMC-containing sample. However, since the dried products are intermediate products, this is not a problem after dissolution. Regarding the crystallinity of the formulations, the amorphous characteristic typical of polymers and spray-dried D-TRE prevailed. This, coupled with the high encapsulation efficiency and thermodynamic solubility values, resulted in a rapid and high solubility and dissolution rate.

The products dissolved in water and adjusted to a given concentration met the requirements of nasal drug delivery systems, as they have a sufficiently low viscosity for dosing, as well as a moderate level of mucoadhesion. Regarding the dynamic viscosity and mucoadhesive strength values, they are also suitable for proper administration and residing in the nasal cavity for approximately 15 to 20 min. The applied polymers did not hinder either the drug release or the absorption. The developed prototype formulations fulfilled the criteria: after a solubilization to a large extent, they have a rapid drug release and absorption profile, which can contribute to increasing the bioavailability via the nasal route and potentially to the uptake of the active substances into the central nervous system.

## 4. Materials and Methods

### 4.1. Materials

Vinpocetine (VP, ethyl-apovincaminate) was applied as a model active substance purchased from Sigma-Aldrich Co., Ltd. (Budapest, Hungary). The Soluplus^®^ (SP, poly(vinyl caprolactam)–poly(vinyl acetate)–poly(ethylene glycol) graft co-polymer (PCL-PVAc-PEG)) was kindly gifted from BASF GmbH (Hannover, Germany), and Poloxamer 188 (P 188, poly(ethylene glycol)-block-poly(propylene glycol)-block-poly(ethylene glycol) (PEG-PPG-PEG)) was also acquired from Sigma-Aldrich Co., Ltd. D-trehalose dihydrate (D-TRE), chitosan (Chit, <100 kDa), sodium hyaluronate (Hya, low molecular weight, 20–40 kDa), hydroxypropyl methylcellulose (HPMC, average molecular weight: 10 kDa, viscosity of 80–120 cP, 2% in water (20 °C)); type III mucin and materials for the simulated nasal electrolyte solution (SNES) were also acquired from Sigma-Aldrich Co., Ltd. The composition of SNES was the following: 8.77 g/L of sodium chloride, 2.98 g/L of potassium chloride and 0.59 g/L of anhydrous calcium chloride in 1000 mL of purified water, adjusted to a pH of 5.6.

### 4.2. Quantitative Analysis of Vinpocetine

The determination of VP concentration was performed with high-performance liquid chromatography (HPLC) using an Agilent 1260 Infinity (Agilent Technologies, Santa Clara, CA, USA) instrument. At the stationary phase, a Kinetex^®^ C18 column (5 µm, 150 mm × 4.6 mm (Phenomenex, Torrance, CA, USA)) was used. In total, 10 µL of the samples was injected to determine the concentration of VP. At mobile phases, a 1.54% *w*/*v* ammonium-acetate solution (A) and acetonitrile (B) were applied in a 40:60 ratio. The separation was performed with isocratic elution for 7 min at 40 °C with a flow rate of 1 mL/min. Chromatograms were detected at 280 ± 4 nm using a UV-Vis diode array detector. The chromatograms were evaluated using ChemStation B.04.03. Software (Agilent Technologies, Santa Clara, CA, USA). The retention time of VP was found at 5.83 min. The limit of detection (LOD) and limit of quantification (LOQ) of VP were 6.31 and 19.11 ppm, respectively. The calibration was performed between 20 and 100 µg/mL where the determined coefficient of linearity (R^2^) value was 0.9997.

### 4.3. Optimization of VP-Loaded Polymeric Micelles

The optimization of VP-loaded polymeric micelles was executed via a 2^3^ factorial design, where the effect of the amount of the applied polymers was investigated on the nanoparticle characteristics (Z-average and polydispersity index) of the polymeric micelles. The independent variables were investigated at 3 levels as it can be seen in Table 8.

In the case of the formulation methodology, SP and Poloxamer 188 were dissolved at determined amounts in 75 mL of purified water. A 25 mL VP solution was prepared in ethanol with a concentration of 1 mg/mL. The solutions were mixed; then, the system was incubated under constant stirring (1000 rpm) at ambient temperature. Finally, 5.0 g of D-TRE was dissolved in the solution. The formulation was produced with a Büchi Nano Spray Dryer equipped with a small nebulizer (Büchi Nano Spray Dyer B-90 HP, Büchi, Flawil, Switzerland). Based on preliminary experiments, the following settings were applied: inlet temperature—100 °C, aspirator capacity—100%, airflow rate—115 mL/min and pump rate—20%. The yield of the formulations was between 87 and 89% on average. 

To investigate the effect of the composition, the quadratic response surface was analyzed, and a second-order polynomial model was constructed using TIBCO Statistica^®^ 13.4 (Statsoft Hungary, Budapest, Hungary) and the relationship of the variables on the response is described via the following second-order equation:

(3) $$Y=\beta_{0}+\beta_{1}x_{1}+\beta_{11}x_{1}^{2}+\beta_{2}x_{2}+\beta_{22}x_{2}^{2}$$

where *Y* is the response variable; *β*_0_ is a constant; *β*_1_ and *β*_2_ are linear coefficients; and β_11_ and *β*_22_ are quadratic coefficients. The analysis of variance (ANOVA) statistical analysis was carried out and the results were evaluated in harmony with their *p*-value when we considered a variable significant if *p* was less than 0.05 at the 95% confidence level. Response surface plots for the polydispersity index and Z-average in the form of contour plots were plotted according to the regression model by keeping one variable at the center level.

### 4.4. Formulation of VP-Loaded Nano-Spray-Dried Polymeric Micelles

VP_Chit: 200 mg of chitosan was dissolved in 75 mL of acidic (using 1 n hydrochloric acid) purified water (pH = 5.5) followed by the dissolution of 300 mg of SP and 250 mg of Poloxamer 188. A 25 mL VP solution was prepared in ethanol with a concentration of 1 mg/mL. The solutions were mixed; then, the system was incubated under constant stirring (1000 rpm) at ambient temperature. Finally, 5.0 g of D-TRE was dissolved in the solution. 

VP_HyA: 0.5 g of HyA was dissolved in 75 mL of cold (4 °C) purified water. From this point, the same formulation was applied as in the case of VP_Chit.

VP_HPMC: 0.5 g of HPMC was placed evenly on the surface of 75 mL of purified water. After complete hydration and dissolution, the same formulation was applied as before.

The formulation of all 3 types of batches was produced with a Büchi Nano Spray Dryer equipped with a small nebulizer (Büchi Nano Spray Dyer B-90 HP, Büchi, Flawil, Switzerland). Based on preliminary experiments, the following settings were applied: inlet temperature—100 °C, aspirator capacity—100%, airflow rate—115 mL/min and pump rate—20%. The yield of the formulations was between 82 and 85% on average. 

For the liquid-state investigations, the formulations were dissolved in purified water according to the drug content. The target concentration of VP was 250 µg/mL. The dispersed samples were checked for drug content before each measurement via HPLC. 

### 4.5. Solid-State Characterization of the Optimized Formulations

#### 4.5.1. Determination of Drug Content

To determine the required amount of spray-dried mass to acquire the target concentration, drug content was measured. At first, samples were measured precisely, followed by the dispersion in 2 mL of a methanol–purified-water mixture in a 1:1 ratio (*v*/*v*). The powders were dissolved under constant stirring (30 min, 1200 rpm). The solutions were filtered through a 0.22-µm-pore-sized polyether sulfone (PES) membrane. The concentration of VP in the samples was determined via HPLC. VP mass was calculated from the concentration acquired with the HPLC measurement. The drug content was expressed as the mass of VP divided by the initial measured mass of the samples. All measurements were carried out in triplicate with individual batches (*n* = 3), and the results are expressed as the average ± SD.

#### 4.5.2. Particle Size Measurement via Laser Diffraction

In order to determine the particle size and particle size distribution of the nano-spray-dried formulation, laser diffraction was used (Malvern Mastersizer Scirocco 2000, Malvern Instruments Ltd., Worcestershire, UK), utilizing the dry dispersion unit. Approximately 0.1–0.3 g of products was loaded into the feeding tray. The dispersion air pressure was 3.0 bar and a vibration feed of 75% was used. The particle size distribution was characterized by the D[0.1]—10% of the volume distribution is below this value; D[0.5]—the volume median diameter; D[0.9]—90% of the volume distribution is below this value; D[2,3]—Sauter mean diameter; and D[3,4]—De Brouckere mean diameter and the calculated Span (as the width of the distribution) values. All measurements were carried out in triplicate with individual batches (n = 3), and the results are expressed as the average ± SD. Span was calculated via the following equation:

(4) $$\mathrm{Span}=\frac{D\left[0.9\right]-D\left[0.1\right]}{D\left[0.5\right]}$$

#### 4.5.3. Morphology Characterization via Scanning Electron Microscopy

The morphology of the nano-spray-dried formulations was characterized with scanning electron microscopy (SEM) (Hitachi S4700, Hitachi Scientific Ltd., Tokyo, Japan). An air pressure of 1.3–13.1 mPa was applied with a high voltage and amperage of 10 kV and 10 mA, respectively. To form the sputter-coated samples conductive with gold–palladium, a high-vacuum evaporator and argon atmosphere were used (Bio-Rad SC 502, VG Microtech, Uckfield, UK). The thickness of the gold–palladium coating was 10 nm. 

#### 4.5.4. X-ray Powder Diffraction Study

The crystalline structure of the nano-spray-dried products was characterized with X-ray powder diffraction (XRPD) using a Bruker D8 Advance X-ray diffractometer (Bruker AXS GmbH, Karlsruhe, Germany) with Cu K λI radiation (λ = 1.5406 Å) and a VANTEC-1 detector. In total, 40 kV of voltage and 40 mA of amperage were used during the measurements. The angular range was 3° to 4° 2θ with a step time of 0.1 s and a step size of 0.007°. The manipulations and evaluations were carried out using EVA Software (EVA Software Solutions, A223, Mumbai, India).

### 4.6. Liquid-State Characterization of the Optimized Formulations

#### 4.6.1. Dynamic Light Scattering Measurements

The micelle size, expressed as the average hydrodynamic diameter (D_H_), the micelle size distribution (PdI, polydispersity index) and the zeta potential were measured via the Malvern Nano ZS Zetasizer (Malvern Instruments, Worcestershire, UK) based on dynamic light scattering. The measurements took place in the target concentration of 250 µg/mL of VP. The samples were placed in folded capillary cells, and the measurements were carried out at 25 °C with a refractive index of 1.650. All measurements were carried out in triplicate with individual batches (n = 3), and the results are expressed as the average ± SD.

#### 4.6.2. Determination of Encapsulation Efficiency

The encapsulation efficiency (EE%) of the nano-spray-dried micelles was determined via the indirect method [24]. The VP-loaded micelles were separated from the aqueous media via centrifugation using a Hermle Z323 K high-performance refrigerated centrifuge (Hermle AG, Gosheim, Germany) at 17,000 rpm and 4 °C for 45 min. The clear supernatant was diluted 5-fold with methanol, then quantitative measurements were carried out via HPLC. All measurements were carried out in triplicate with individual batches (*n* = 3), and the results are expressed as the average ± SD. The encapsulation efficiency was calculated via this equation:

(5) $$\mathrm{EE}\%=\frac{\mathrm{initial}\;\mathrm{VP}\;\left(\mathrm{mg}\right)-\mathrm{measured}\;\mathrm{VP}\;\left(\mathrm{mg}\right)}{\mathrm{initial}\;\mathrm{VP}\;\left(\mathrm{mg}\right)}\times 100$$

#### 4.6.3. Determination of Thermodynamic Solubility

To determine the thermodynamic solubility of the formulations, 0.5 mL of purified water was measured into vials and the formulations were dissolved until visible saturation [10]. Covered with parafilm, the saturated solutions were under constant stirring with a magnetic stirrer for 72 h at ambient temperature. Then, the solutions were filtered through a 0.22-µm-pore-sized PES membrane. The passed-through VP amount was measured via HPLC. All measurements were carried out in triplicate with individual batches (*n* = 3), and the results are expressed as the average ± SD.

#### 4.6.4. Viscosity Measurement

The viscosity of the dispersed formulations compared to pure mucoadhesive excipient solutions was measured with a Physica MCR 302 rheometer (Anton Paar, Graz, Austria). The measuring device was of the cone and plate type (diameter: 25 mm, cone angle: 1°, gap height in the middle of the cone: 0.05 mm). Flow curves of the emulsions were plotted from a 0.1 to 100 s^−1^ shear rate. The viscosity of the sample was determined at a 50 s^−1^ shear rate using the interpolation function of the RheoCompass Software (Anton Paar GmbH, Ashland, VA, USA) of the instrument. Three parallel measurements were performed. Results are expressed as means ± SD (*n* = 3).

### 4.7. Nasal Applicability Studies

#### 4.7.1. In Vitro Mucoadhesion Study

To investigate the in vitro mucoadhesivity of the formulations, the tensile tests were performed using a TA-XT Plus Texture analyser (Metron Ltd., Budapest, Hungary) equipped with a 5 kg load cell and a cylinder probe, which had a diameter of 1 cm. The dispersed formulations were placed in contact with a filter paper disc with a 25 mm diameter wetted with 50 µL of an 8% *w*/*w* porcine mucin (type III) dispersion in SNES (pH 5.6). In total, 20 µL of the dispersed formulations was attached to the filter paper fixed on a cylinder probe and placed in contact with the mucin dispersion. A 2500 mN preload was used for 3 min; then, the cylinder probe with the dispersed formulations was moved upwards at a prefixed speed of 2.5 mm × min^−1^ to separate the attaching surfaces. Adhesive force (F, mN) and adhesive work (A, mN × mm) were applied for the evaluation of the mucoadhesivity. Five parallel measurements were performed from individual batches and the results are expressed as means ± SD [28].

#### 4.7.2. In Vitro Drug Release Study

The drug release study was performed using the modified paddle method. Dispersed formulations were placed in dialysis tubes (Spectra/Por^®^ Dialysis Membrane with a 12–14 kD MWCO (Spectrum Laboratories Inc., Rancho Dominguez, CA, USA)). The tubes were placed in 100 mL of SNES, which functioned as a dissolution media. The measurement was carried out at 35 °C under 100 rpm of paddle rotation. At predetermined time points, 0.5 mL of aliquots was taken up to 60 min and quantification was performed via HPLC. Three parallel measurements were performed from individual batches and the results are expressed as means ± SD.

#### 4.7.3. Determination of Drug Release Kinetics

For the determination of drug release kinetics, six different mathematical models (zero-order, first-order, second order, Korsmeyer–Peppas, Higuchi, Hixon–Crowell) were fitted with the obtained cumulative drug release vs. time curves to describe the kinetics. The evaluation was based on the comparison of the obtained regression coefficients (R^2^).

#### 4.7.4. In Vitro Nasal Diffusion Study

The in vitro nasal diffusion study of DXM from the nasal cavity was performed in a modified Side-Bi-Side^®^ horizontal diffusion cell, where a cellulose membrane impregnated with isopropyl myristate was applied with a surface of 0.785 cm^2^. Both the donor and acceptor cell volumes were 9.0 mL among which the diffusion was investigated at 35 °C. The donor concentrations corresponded to the previously set up 0.5 mg/mL. The donor phase consisted of SNES and the acceptor phase was a pH 7.4 PBS. Sampling from the acceptor phase was performed at assigned time points and the VP concentration was measured via HPLC. The flux (J) was calculated from the quantity of VP that permeated through the membrane, divided by the surface membrane insert and the duration of the experiment (µg/cm^2^/h). The permeability coefficient (*K_p_*) was determined from *J* and the drug concentration in the donor phase (*C_d_* (µg/cm^3^)) as seen in the following equation [28]:

(6) $$K_{p}\left[\frac{cm}{h}\right]=\frac{J}{C_{d}}$$

## 5. Conclusions

In summary, it can be claimed that the nano-spray-drying technique can be perfectly used to produce polymeric micelles, which alone, but also when supplemented with nasal mucoadhesive polymer excipients, can add value to active substances with a poor solubility and permeation profile. The formulations can also increase patient adherence due to the ease of administration and they can be utilized in the intranasal therapy of dementia and related cognitive diseases.

## Figures and Tables

**Table 1:** Results from the 2-factor, 3-level factorial design. Results are presented as average ± SD (*n* = 3).

Run No.	Soluplus^®^ (mg)	Poloxamer 188 (mg)	Z-Average (nm)	PdI
1	200.0	200.0	69.07 ± 11.4	0.521 ± 0.017
2	200.0	250.0	79.31 ± 5.7	0.354 ± 0.028
3	200.0	300.0	74.69 ± 13.2	0.321 ± 0.009
4	250.0	200.0	76.04 ± 3.8	0.460 ± 0.054
5	250.0	250.0	127.7 ± 4.5	0.124 ± 0.041
6	250.0	300.0	243.7 ± 2.9	0.379 ± 0.031
7	300.0	200.0	135.6 ± 5.9	0.295 ± 0.018
8	300.0	250.0	121.3 ± 1.8	0.039 ± 0.007
9	300.0	300.0	304.5 ± 26.7	0.467 ± 0.075

**Table 2:** D[0.1], D[0.5] and D[0.9] particle size values characterizing initial VP and the nano-spray-dried formulations containing the applied low-concentration mucoadhesive excipients. Span was calculated based on these particle size values. Results are expressed as average ± SD (*n* = 3). Abbreviations: VP, initial vinpocetine; VP_Chit, chitosan (Chit)-containing nano-spray-dried formulation; VP_HyA, hyaluronic acid (HyA)-containing nano-spray-dried formulation; VP_HPMC, hydroxypropyl methylcellulose (HPMC)-containing nano-spray-dried formulation.

	VP	VP_Chit	VP_HyA	VP_HPMC
**D[0.1] (µm)**	9.24 ± 1.37	2.12 ± 0.05	1.23 ± 0.10	2.79 ± 0.31
**D[0.5] (µm)**	41.23 ± 2.13	7.34 ± 0.19	7.08 ± 0.25	7.15 ± 0.07
**D[0.9] (µm)**	173.87 ± 13.58	9.15 ± 1.12	8.73 ± 1.41	9.94 ± 1.51
**Span**	3.99 ± 0.17	0.96 ± 0.04	1.06 ± 0.08	1.02 ± 0.14

**Table 3:** The theoretical and measured VP active substance content in 1000 mg of the nano-spray-dried powders. Results are expressed as average ± SD (n = 3).

	VP_Chit	VP_HyA	VP_HPMC
**m_VP_ theoretical (mg/g)**	4.33	4.15	4.15
**m_VP_ measured (mg/g)**	4.12 ± 0.08	4.07 ± 0.05	4.09 ± 0.09

**Table 4:** Average hydrodynamic diameter (D_H_), polydispersity index (PdI) and zeta potential (ζ) values of the nano-spray-dried formulations after dispersion in water to the target concentration of 250 µg/mL. Measurements took place via dynamic light scattering and zeta potential measurements. All data are presented as means ± SD from three individual batches.

	VP_Chit	VP_HyA	VP_HPMC
**D_H_ (nm)**	133.6 ± 4.8	110.8 ± 2.5	102.5 ± 3.1
**PdI**	0.240 ± 0.016	0.265 ± 0.007	0.246 ± 0.011
**ζ (mV)**	27.16 ± 2.16	−11.0 ± 1.34	−2.05 ± 0.39

**Table 5:** Encapsulation efficiency (EE%) measured via the indirect method of the nano-spray-dried formulations and the thermodynamic solubility of raw VP and the formulations via the saturation method. Quantification was performed via HPLC. All data are presented as means ± SD from three individual batches.

	VP	VP_Chit	VP_HyA	VP_HPMC
**EE (%)**	-	91.35 ± 1.40	86.14 ± 2.32	85.44 ± 3.04
**S_25°C_ (µg/mL)**	2.39 ± 0.37	713.81 ± 23.14	658.11 ± 17.40	661.76 ± 37.65

**Table 6:** Obtained kinetic parameters of the nano-spray-dried VP-loaded polymeric micelles compared to the initial VP.

Model		VP	VP_Chit	VP_HyA	VP_HPMC
Zero order	k_0_ (µg/min)	0.1188	2.154	2.117	1.9128
	R^2^	0.7971	0.7256	0.7001	0.8361
	t_0.5_ (min)	420.88	23.21	23.62	26.14
First order	k_1_ (min^−1^) × 10^−3^	0.83	44.36	38.23	37.28
	R^2^	0.7404	0.8601	0.8435	0.9466
	t_0.5_ (min)	839.76	15.62	18.13	18.59
Second order	k_2_ (µg^−1^ min^−1^) × 10^−5^	0.85	237.24	176.82	137.13
	R^2^	0.747	0.9825	0.9681	0.9931
	t_0.5_ (min)	1154.80	4.15	4.15	8.93
Korsmeyer–Peppas	k_K-P_ (min^−n^)	2.0044	0.7571	0.6794	0.9219
	n	0.4796	0.5367	0.4754	0.5638
	R^2^	0.9541	0.9370	0.9021	0.9839
	t_0.5_ (min)	230.76	56.88	139.53	23.89
Higuchi	k_H_ (µg min^−1/2^)	0.8241	15.385	15.299	12.989
	R^2^	0.9116	0.9944	0.9834	0.9937
	t_0.5_ (min)	3682.02	10.56	10.68	14.82
Hixson–Crowell	k_H-C_ (µg^1/3^ min^−1^) × 10^−3^	3.004	96.69	91.98	69.93
	R^2^	0.8381	0.9270	0.8875	0.97745
	t_0.5_ (min)	318.68	9.90	10.41	13.69
Best fit		Korsmeyer–Peppas	Higuchi	Higuchi	Higuchi

**Table 7:** Calculated parameters reflecting the permeability profile of initial VP compared to the nano-spray-dried formulations. Flux (J), permeability coefficient (K_p_) and the apparent permeability (P_app_). Data are presented as average ± SD (*n* = 3).

	VP	VP_Chit	VP_HyA	VP_HPMC
**J (µg/cm^2^)**	14.5 ± 3.1	237.6 ± 19.5	215.9 ± 21.2	197.77 ± 14.6
**K_p_ (cm/h)**	0.029	0.4752	0.4318	0.3955
**P_app_ (cm/s) × 10^−3^**	0.145	2.376	2.159	1.97

**Table 8:** The investigated polymer amounts at 3 levels in the optimization process of VP-loaded polymeric micelles.

**Independent Factors**	**Levels**		
	**−1**	**0**	**+1**
Soluplus^®^ (mg)	200.0	250.0	300.0
Poloxamer 188 (mg)	200.0	250.0	300.0

## Data Availability

The data presented in this study are available on request from the corresponding author.

## References

[1] Lauffleur F., Bauer B. (2021). Progress in nasal drug delivery systems. Int. J. Pharm..

[2] Rabiee N., Ahmadi S., Afshari R., Khalaji S., Rabiee M., Bagherzadeh M., Fatahi Y., Dinarvand R., Tahriri M., Tayebi L. (2020). Polymeric Nanoparticles for Nasal Drug Delivery to the Brain: Relevance to Alzheimer’s Disease. Adv. Ther..

[3] Shim S., Yoo H.S. (2020). The application of mucoadhesive chitosan nanoparticles in nasal drug delivery. Mar. Drugs.

[4] Illum L. (2003). Nasal drug delivery—Possibilities, problems and solutions. JCR.

[5] Illum L. (2016). Nasal drug delivery—Recent developments and future prospects. JCR.

[6] Owen S.C., Chan D.P., Shoichet M.S. (2012). Polymeric micelle stability. Nano Today.

[7] Ghezzi M., Pescina S., Padula C., Santi P., Del Favero E., Cantù L., Nicoli S. (2021). Polymeric micelles in drug delivery: An insight of the techniques for their characterization and assessment in biorelevant conditions. JCR.

[8] Hwang D., Ramsey J.D., Kabanov A.V. (2020). Polymeric micelles for the delivery of poorly soluble drugs: From nanoformulation to clinical approval. Adv. Drug Deliv. Rev..

[9] Suzuki K., Yoshizaki Y., Horii K., Murase N., Kuzuya A., Ohya Y. (2022). Preparation of hyaluronic acid-coated polymeric micelles for nasal vaccine delivery. Biomater. Sci..

[10] Sipos B., Csóka I., Budai-Szűcs M., Kozma G., Berkesi D., Kónya Z., Balogh G.T., Katona G. (2021). Development of dexamethasone-loaded mixed polymeric micelles for nasal delivery. Eur. J. Pharm. Sci..

[11] Chaudhari S.P., Shinde P.U. (2020). Formulation and characterization of tranylcypromine loaded polymeric micellar in-situ nasal gel for treatment of depression. Technology.

[12] Sipos B., Bella Z., Gróf I., Veszelka S., Deli M.A., Szűcs K.F., Sztojkov-Ivanov A., Ducza E., Gáspár R., Kecskeméti G. (2022). Soluplus^®^ promotes efficient transport of meloxicam to the central nervous system via nasal administration. Int. J. Pharm..

[13] Pandey J., Shankar R., Kumar M., Shukla K., Kumari B. (2020). Development of nasal mucoadhesive microspheres of granisetron: A potential drug. Drug Res..

[14] Kazi-Chishti M., Shaikh J., Chishti N., Dehghan M.H. (2023). Nasal mucoadhesive in situ gelling liquid crystalline fluid precursor system of polyene antibiotic for potential treatment of localized sinuses aspergillosis post COVID infection. J. Dispers. Sci. Technol..

[15] Trenkel M., Scherließ R. (2021). Nasal powder formulations: In-vitro characterisation of the impact of powders on nasal residence time and sensory effects. Pharmaceutics.

[16] Kang M.L., Cho C.S., Yoo H.S. (2009). Application of chitosan microspheres for nasal delivery of vaccines. Biotechnol. Adv..

[17] Tomono T., Yagi H., Ukawa M., Ishizaki S., Miwa T., Nonomura M., Igi R., Kumugai H., Miyata K., Tobita E. (2020). Nasal absorption enhancement of protein drugs independent to their chemical properties in the presence of hyaluronic acid modified with tetraglycine-L-octaarginine. Eur. J. Pharm. Biopharm..

[18] Bentley K., Stanton R.J. (2020). Hydroxypropyl methylcellulose-based nasal sprays effectively inhibit in vitro SARS-CoV-2 infection and spread. Viruses.

[19] Protopapa C., Siamidi A., Pavlou P., Vlachou M. (2022). Excipients Used for Modified Nasal Drug Delivery: A Mini-Review of the Recent Advances. Materials.

[20] Park H.-Y., Kweon D.-K., Kim J.-K. (2023). Molecular-weight-dependent hyaluronic acid permeability and tight junction modulation in human buccal TR146 cell monolayers. Int. J. Biol. Macromol..

[21] Niu J., Yuan M., Zhang Z., Wang L., Fan Y., Liu X., Liu X., Ya H., Zhang Y., Xu Y. (2022). Hyaluronic acid micelles for promoting the skin permeation and deposition of curcumin. Int. J. Nanomed..

[22] Dodane V., Khan M.A., Merwin J.R. (1999). Effect of chitosan on epithelial permeability and structure. Int. J. Pharm..

[23] Sadeghi A.M.M., Dorkoosh F.A., Avadi M.R., Weinhold M., Bayat A., Delie F., Gurny R., Larijani B., Refiee-Tehrani M., Junginger H.E. (2008). Permeation enhancer effect of chitosan and chitosan derivatives: Comparison of formulations as soluble polymers and nanoparticulate systems on insulin absorption in Caco-2 cells. Eur. J. Pharm. Biopharm..

[24] McInnes F.J., O’Mahony B., Lindsay B., Band J., Wilson C.G., Hodges L.A., Stevens H.N.E. (2007). Nasal residence of insulin containing lyophilised nasal insert formulations, using gamma scintigraphy. Eur. J. Pharm. Sci..

[25] Yang Y.T., Chen C.T., Yang J.C., Tsai T. (2010). Spray-dried microparticles containing polymeric micelles encapsulating hematoporphyrin. AAPS J..

[26] Sipos B., Csóka I., Ambrus R., Schelz Z., Zupkó I., Balogh G.T., Katona G. (2022). Spray-dried indomethacin-loaded polymeric micelles for the improvement of intestinal drug release and permeability. Eur. J. Pharm. Sci..

[27] Pepić I., Lovrić J., Filipović-Grčić J. (2013). How do polymeric micelles cross epithelial barriers?. Eur. J. Pharm. Sci..

[28] Sipos B., Csóka I., Szivacski N., Budai-Szűcs M., Schelcz Z., Zupkó I., Szabó-Révész P., Volk B., Katona G. (2021). Mucoadhesive meloxicam-loaded nanoemulsions: Development, characterization and nasal applicability studies. Eur. J. Pharm. Sci..

